# The Potential of Adaptive Design in Animal Studies

**DOI:** 10.3390/ijms161024048

**Published:** 2015-10-12

**Authors:** Arshad Majid, Ok-Nam Bae, Jessica Redgrave, Dawn Teare, Ali Ali, Daniel Zemke

**Affiliations:** 1 Sheffield Institute for Translational Neuroscience, University of Sheffield, Sheffield S10 2HQ, UK; E-Mails: Jessica.Redgrave@sth.nhs.uk (J.R.); Ali.Ali@sth.nhs.uk (A.A.); zemkedan@gmail.com (D.Z.); 2 College of Pharmacy Institute of Pharmaceutical Science and Technology, Hanyang University, Ansan 426-791, Korea; E-Mail: onbae@hanyang.ac.kr; 3 School of Health and Related Research, University of Sheffield, Sheffield S10 2HQ, UK; E-Mail: m.d.teare@sheffield.ac.uk

**Keywords:** adaptive design, animal studies, preclinical research

## Abstract

Clinical trials are the backbone of medical research, and are often the last step in the development of new therapies for use in patients. Prior to human testing, however, preclinical studies using animal subjects are usually performed in order to provide initial data on the safety and effectiveness of prospective treatments. These studies can be costly and time consuming, and may also raise concerns about the ethical treatment of animals when potentially harmful procedures are involved. Adaptive design is a process by which the methods used in a study may be altered while it is being conducted in response to preliminary data or other new information. Adaptive design has been shown to be useful in reducing the time and costs associated with clinical trials, and may provide similar benefits in preclinical animal studies. The purpose of this review is to summarize various aspects of adaptive design and evaluate its potential for use in preclinical research.

## 1. Introduction

Preclinical studies are an essential component of medical research, providing useful data on the efficacy and safety of prospective treatments prior to clinical trials. Many use animal subjects, and may be limited by cost and ethical concerns. Increasing efficiency, without compromising the integrity of the research, is therefore desirable. One option is to adopt strategies used to increase the efficiency of clinical trials. Traditional clinical trials typically test all potential variables that may affect the results, however not all combinations of variables are equally informative [1]. Allocating test subjects to potentially uninformative or ineffective treatment arms is an inefficient use of resources and has ethical implications as well. Furthermore, statistical analysis is often performed at the conclusion of the study, and is therefore limited by the quality of the data collected. These issues apply to animal studies as well. Planning of clinical trials often involves design optimization, in which mathematical models are used to determine the experimental design which will produce the maximum amount of useful information for the lowest cost [1]. In many cases, design optimization is performed only during the planning stage, and the optimal design is subsequently used without modification. An alternative strategy, however, is to allow changes to be made during the study based on intermediate results or new external data. This process is known as adaptive design, the benefits of which include lower cost, reduced development time, and improved care to test subjects. Although currently used primarily in clinical trials, it is possible that preclinical studies could also benefit from its use. Adaptive design is not always advantageous, however, and its increasing use has been the focus of some concern. In this review, various aspects of adaptive design—including its advantages and disadvantages as well as its logistic, financial, and regulatory implications—will be discussed with respect to its potential application to animal studies.

## 2. Adaptive Design and Its Types

Traditional clinical trials utilize predetermined protocols that are not intended to be altered. In practice, however, unplanned changes are sometimes made in response to nuisance factors or serious negative consequences, which may be considered a form of adaptive design. In this review, however, the term adaptive design will be used to refer to studies in which design modifications are explicitly allowed or even pre-planned. Numerous studies incorporating elements of adaptive design can be found in the literature, which has led to some confusion over what adaptive design really is. The Pharmaceutical Research and Manufacturers of America (PhRMA) defines an adaptive design study as one that allows modifications based on accumulating data without compromising the validity or integrity of the study [2]. The United States Food and Drug Administration (FDA) defines adaptive design as a prospectively planned opportunity for modification based on interim data [3]. PhRMA further specifies that adaptations should be included by design rather than *ad hoc*, whereas the FDA recommends pre-planned modifications, but allows other changes to be made before unblinding of the data [4,5]. *Ad hoc* modifications are more flexible and reflect actual clinical practice, in which continual adjustments to patient care are made in order to produce the best possible outcome; however they are more prone to misuse or overuse [4].

A wide variety of design modifications exist (Table 1), including modification of randomization schedules, stopping a trial early, sample size re-estimation, dropping inferior treatment arms, modification of dosage levels, adaptations based on prognostic biomarkers, enrichment of the study population for patients mostly likely to benefit from treatment, switching to an alternative treatment, modifications to the hypothesis, changing endpoints, changes in statistical analysis, or a combination of multiple modifications. The types of adaptive design modifications have been covered previously and will not be described in detail here [4,5,6,7]. The FDA classifies adaptive designs as either well understood or less understood [3]. Well understood designs include changing eligibility criteria, adaptations to maintain study power, adaptations based on outcomes unrelated to efficacy, early study termination, and changes to data analysis. These designs have established decision-making procedures and properly account for any adaptations made, therefore no statistical modifications are typically necessary. Examples of less understood designs include adaptive dose selection, adaptive randomization, sample size re-estimation, changing the patient population, changing endpoints, non-inferiority studies, and studies with multiple modifications. These designs rely on analysis of unblinded data, which may introduce bias or affect interpretation of the final analysis, and have an increased risk of Type I error (false positives). Adaptive designs may also be classified as learning stage (such as determining efficacy and toxicity) or confirmatory stage, or combine both in seamless combined Phase I/IIa or Phase IIb/III trials [5]. Learning stage designs are generally more accepted, require less regulatory approval, and are concerned more with Type II error (false negatives) than Type I. Confirmatory stage designs, particularly those that are less understood, typically require more justification. Seamless designs increase efficiency by using the same population for both stages. Since data from the first stage influences the design of the second stage, however, corrections for bias must be made.

**Table 1:** Modifications used in adaptive design studies.

Modification	Description
Adaptive randomization	Interim results used to assign subjects into treatment groups.
	Allows greater allocation of resources to most informative groups.
Sample size re-estimation	Changes to group size based on interim statistical power calculations.
	Used to determine if studies can be stopped early or need to be extended.
Population enrichment	Selective enrollment of subjects most likely to benefit from treatment.
	Improves detection of small effects and eliminates confounding variables.
Adaptive dose finding	Alteration of dosage for each subject based on results from the previous subject.
	Allows rapid determination of optimal dose and minimization of adverse effects.
Treatment switching	Administration of alternative treatments to subjects experiencing adverse effects or in ineffective treatment arms.
	Ensures subjects receive best possible care.
Dropping treatment groups	Elimination of unsuccessful treatment arms.
	Allows reduced sample size or reassignment of subjects to other groups.
Alteration of endpoints	Changes to predetermined stop points.
	Allows study to be stopped early if significant benefits or adverse effects are seen.
Alterations to hypothesis	Changes to initial hypothesis based on unexpected results or new external information.
	May be used to change hypothesis from one of superiority to non-inferiority.
Alterations to statistical analysis	Changes made to statistical procedures to accommodate design modifications or unexpected variables.

Several of the adaptive modifications mentioned above could potentially be used to increase efficiency and improve results in preclinical studies involving animals. Sample size re-estimation, for example, may indicate that fewer animals are required to achieve statistical significance for treatments with strong effects, reducing both costs and the number of animals exposed to distressing or painful procedures. Alternatively, more animals may be needed to detect smaller effects. Dropping an ineffective treatment group could reduce the number of animals used or allow them to be reassigned to other groups, thus increasing statistical power. In addition, treatment groups are often not equally informative. Therefore, the common practice of equal group sizes is an inefficient use of resources. Adaptive randomization could allow group sizes based on the probability of detecting an effect. There are, however, several issues with introducing such modifications, which will be discussed in later sections.

## 3. Adaptive Design Methods

Adaptive design relies heavily on statistical models and simulation. Different modifications require different sets of calculations, and multiple mathematical models have been developed for some modification types. As a result, a wide variety of competing models exist in the literature. Standardized procedures exist in some cases, but statistical methods for less understood designs are not well established at this time [4]. Most models, however, share common features. The first step is the formation of the initial model [1]. All controllable parameters must be identified and mathematically expressed. As such, the parameters must be quantifiable. For each parameter, the probability of a particular outcome is calculated, which may be based on prior studies, pilot tests, or general consensus. The parameter is then defined by the distribution of the collected probabilities for all possible outcomes, and a statistical model is created using the probability distributions of all the parameters. Different study designs can then be compared by calculating the average payoff for all possible outcomes of a particular design, and the design with the highest average payoff is selected as the initial optimal design. At specified intervals, data collected using the current design is used to re-evaluate its utility, and the design is updated as necessary. This process is repeated until predefined stopping points are reached, such as a certain number of intervals or until a parameter reaches a specific value [1]. An exception to this process is modifications made after data collection is complete, such as changing the statistical methods used for the final analysis.

A 2006 review of 60 adaptive design studies found that sample size re-estimation was the most frequently used modification [8]. Changing samples sizes allows early termination of a trial in the event of significant benefits, no benefit, or unforeseen negative consequences. In many cases, sample size adjustment uses unblinded data. It has been determined, however, that unblinding is not required for efficient sample size calculation [9]. Adding in the option for early termination, on the other hand, increases variability. Another common use for adaptive design is in dose response studies, for which many different methods are available. Design-focused approaches make modifications during the study based on collected data, whereas analysis-focused approaches involve choosing the best statistical methods for analysis [10]. One design-focused approach that is particularly attractive in the clinical setting is the continual reassessment method [11]. In this method, the dose is adjusted after each patient, allowing the maximum tolerable dose to be determined more quickly than in traditional dose escalation studies. Increasing the number of modifications made during a study, however, increases the complexity of the design. In contrast, analysis-focused approaches do not reduce the time or costs associated with a study, but determine the statistical methods most likely to detect a particular effect. Bornkamp *et al.* used computer simulations to compare traditional methods using analysis of variance (ANOVA) with both design-focused and analysis-focused adaptive methods [10]. The simulations included the effects of sample size, dose response profile, number of different doses, and the number of adaptations made. All of the tested methods adequately controlled Type I error. In general, design-focused approaches were best at estimating the dose response, selecting a target dose interval, and detecting clinical relevance; whereas ANOVA was the worst. Many of the adaptive approaches, however, lost their advantage at smaller sample sizes.

## 4. Advantages and Disadvantages of Adaptive Design

Table 2 lists some of the advantages and disadvantages of adaptive design. One of the primary advantages is the potential for increased efficiency. Modifications such as adaptive dose finding, dropping treatment arms, sample size re-estimation, and early trial termination allow a study to be completed more quickly, with fewer subjects, and at lower cost. This accelerates the development of beneficial treatments, which is highly desirable in a clinical setting. Patients with untreatable diseases could benefit greatly from decreased development times, and increased statistical power with smaller sample sizes could be useful in studies of rare diseases or other small trials with limited target populations [5]. Animal studies often have smaller sample sizes and more limited funding than those conducted in humans, and adaptive design could compensate for these problems. The choice of design is important, however, as not all adaptive designs retain power at small sample sizes [10]. In addition, smaller sample sizes increase variability and decrease the ability to detect smaller effects [5]. Although animal studies—particularly those using rodents—generally use inbred lines with reduced variability, this may still be a concern.

**Table 2:** Advantages and disadvantages of adaptive design.

**Advantages**
Decreased treatment development time and study duration
Decreased resource usage and program cost
Increased statistical power at small sample sizes
Improved subject care and minimization of adverse effects
Allows correction for incorrect initial assumptions or unexpected data
Allows incorporation of emerging external data
**Disadvantages**
Not applicable to some types of studies
Promising treatments may be dropped prematurely
Small sample sizes increase variability and reduce detection sensitivity
Increased Type I error
Increased planning, simulation, and analysis required
May require use of unblinded data
Potential for misuse or abuse
Regulatory concerns

Several groups have questioned the common assumption that adaptive designs are always more efficient [12,13,14]. In a comparison of different designs for survival analysis or time to a particular event, adaptive design was more cost effective than traditional group sequential studies at low subject accrual rates, but not at high accrual rates [12]. In addition, group sequential studies were more efficient in terms of average sample number for a given sample size, with equal or greater power. Regardless of sample size, however, time savings associated with adaptive design favored its use when treatment costs prior to enrolling were high relative to the cost per patient within the study. Another group has claimed that for any adaptive design, it is possible to design a similar group sequential study that will accept or reject the null hypothesis earlier and with higher probability [13]. It has also been stated that traditional designs with additional analysis have similar benefits to—and less complexity than—adaptive designs [14]. However, opinions may vary as to which aspects of a design are most important and, therefore, what constitutes an optimal design.

In addition to increased efficiency, there are also ethical benefits to adaptive design. Adaptive dose finding decreases the number of subjects exposed to ineffective or toxic doses and allows a faster transition to safe and effective doses. Similarly, dropping inferior treatment groups allows subjects to be reassigned to ones that are more successful. Adaptive treatment switching, biomarker adaptive strategies, and target population enrichment allow subjects to receive better, more individualized care than by random group assignment. Increased efficiency, however, must be weighed against the cost and ethical implications of poor subject outcome [15]. Ethical considerations are a critical component of animal studies, especially those using higher organisms such as mammals. In particular, the number of animals used and their exposure to pain or distress must be minimized. Adaptive design would allow the required number of animals to be reduced if a significant effect is detected early or potentially painful treatments to be dropped if no effect is seen.

Accordingly, another advantage of adaptive design is its flexibility. Adaptive design allows changes in response to surprising data or new external information [4]. It also allows incorrect assumptions made during planning to be corrected. On the other hand, this flexibility introduces the possibility of misuse. Significant modifications may invalidate the methods used, alter the target population, or introduce bias and variability [4,6]. Promising treatment groups may be inadvertently dropped based on preliminary data [4]. Changing the primary endpoint may alter the hypothesis being tested, which is a cause for concern [16]. It is also possible that modifications may be abused in order to achieve the desired result, especially in the case of unplanned modifications.

Adaptive design also has implications with regard to statistical analysis. A significant issue with making modifications to a study design is the possible introduction of Type I error [4]. For example, dropping a treatment group and reassigning its members to other groups increases statistical power, but increases Type I error as well [17,18]. Preventing Type I error at a pre-specified level of significance may be difficult, leading to incorrect *p* values and unreliable confidence intervals [6]. Error control is thus an important consideration for any statistical procedures to be performed. Modifications may also lead to inconsistency between the hypothesis and the statistical methods used to test it. Another issue is how to analyze combined data when different methods were used for different parts of the study [4]. Introducing changes increases the complexity of the study design and consequently the complexity of the analysis.

There are also other limitations of adaptive design. During design optimization, it is impossible to test every possible model. Therefore, the design chosen might not represent the ideal one [1]. In addition, variables that are not quantifiable cannot be optimized; therefore optimization cannot be applied to certain types of studies. Furthermore, the optimization step only takes previous experiments into account, but whether or not a design is optimal also depends on future experiments. Another limitation is that adaptive design offers no time advantage in long term studies, due to the inability to make adjustments at shorter intervals [4]. It is also not useful when simultaneously testing multiple factors, which makes statistical analysis prohibitively complex. One particular concern is that even blinded interim data may give an indication of results to the investigator and introduce bias [18]. For example, changing group sizes after sample size re-estimation may allow the relative effectiveness of various treatments to be determined [5]. Basing sample size calculations on estimates of nuisance parameters in an internal pilot rather than actual test results is less controversial, but unless the group allocation is masked, it still may be possible to determine treatment effects. Before implementing an adaptive design, the relevant advantages and disadvantages need to be considered in order to determine if it will provide greater benefit than a traditional design.

## 5. Logistic and Regulatory Considerations

Planning and analysis for an adaptive design often requires more time and effort than for a traditional design. It is therefore important to consider if the potential benefits of an adaptive design outweigh the extra work involved. One challenge is the lack of individuals with sufficient understanding of the methods involved, which may lead to increased costs and delayed development if a study is improperly designed [19,20]. The availability of these individuals is therefore an important consideration. External statistical assistance may be required if the investigators themselves do not possess the requisite knowledge, the cost of which may offset any gains to be made. Extensive computations and simulations can be expensive, which may be prohibitive to publicly funded studies which lack the necessary resources and infrastructure [5]. Highly complex studies, therefore, may be better suited to the industrial sector. Although the cost savings from using shorter studies with fewer subjects may be greater than the cost of increased planning and analysis in the clinical setting, the opposite may be true in animal studies, where standard procedures and subject care are not as expensive. Another consideration is the availability of appropriate statistical software [4,9]. Although a few packages exist that can handle the necessary calculations, most statistical software currently in use is inadequate for the task. In addition, statistical models may have to be custom designed for less understood designs without established procedures, leading to the inability to compare studies due to differences in methodology. In some cases, however, model simulations have been constructed that can be performed using free packages such as R [11].

Other design features must also be considered when planning an adaptive design study, for instance the number of interim analyses. More frequent updates increase the speed and efficiency of a study, but less data is available at each stage [1]. Less frequent updates allow each stage to incorporate more data, but are slower and more computationally complex. With optimization at discrete intervals, it may be difficult to acquire complete data at each interval [9]. Continuous optimization methods, on the other hand, require an appropriate infrastructure for real time data capture. It is also important to consider how much modification is acceptable and if it could cause deviation from the theoretical model [4]. The impact of noncompliance with the pre-planned methods on the validity of the study is another consideration. Once the primary endpoint is reached, an important question may be how to test secondary endpoints [16]. In addition, if interim analyses require unblinded data, the possibility of bias must be taken into account. In this case, independent data monitoring may be advisable.

In terms of regulatory concerns, the standards and requirements for review and approval of a study must be considered. Proposed studies may be rejected due to a lack of reviewer understanding of adaptive design [5]. In addition, confusion over what constitutes adaptive design and controversy over less understood designs may lead to mistrust of more established designs [21]. Traditional funding schemes or regulations may also not allow certain modifications, such as changing sample sizes [5]. These concerns are particularly relevant to animal studies. To date, adaptive design has been mainly used in clinical trials; therefore not all reviewers may be familiar with it. Reviewers without a statistical background may become confused by the calculations involved in the planning and analysis of a study. In addition, regulations on the humane use of animal subjects may restrict the ability to make certain modifications. Approval to use animal subjects often requires the number of animals and methods to be specified beforehand. While modifications that reduce animal use or drop harmful procedures would likely be welcome, increasing sample sizes or adding unapproved doses and procedures would need further justification. Changes to animal use regulations may therefore be necessary in order to incorporate adaptive design.

## 6. Examples of Adaptive Design in Animal Studies

In an evaluation of adaptive design strategies for clinical trials, Lewis and Berry tested their applicability to animal studies as well [22]. Results from a traditional study of epinephrine and cardiopulmonary resuscitation prior to defibrillation for cardiac arrest in dogs were examined using various methods of determining endpoints. The original study utilized a sequential design and detected a significant difference in survival rate after 28 animals. Using a Bayesian approach, the study would also have required 28 animals to reach statistical significance. Using two other approaches, however, the results were not significant at the first interim analysis and the number of animals required was increased. Thus, there was no advantage to nontraditional designs in this case. This example, however, was merely a simulation. Little evidence of adaptive design being actively used in animal studies can be found in the current literature. The two examples given below were identified because adaptive methods were specifically mentioned in the abstract. It is possible that other examples exist, but the lack of mention of adaptive design in animal studies indicates that it is not very common.

In a 2012 study, adaptive methods were used in an evaluation of dexamethasone and desoxycorticosterone following intrabronchial challenge with *Staphylococcus aureus* in dogs [23]. In the first stage, an adaptive dose finding strategy was used to determine the optimal dose of *S. aureus* required to produce 80% to 90% mortality. Challenge doses were evaluated at four intervals of four to six animals each and the optimal dose was selected for use in the second stage of the study. Data from both stages was used in the final analysis, although the statistical tests used do not appear to be tailored to an adaptive design. It is also unclear whether the methods used represent actual adaptive design or just sequential dose escalation. In another study, the effects of levetiracetam were investigated in dogs with status epilepticus or acute repetitive seizures [24]. At a planned interval of 10 dogs, the adverse effects of the initial dosage were evaluated. Subsequently, the dose was increased for the remaining nine dogs. Although this study incorporated a planned interval analysis, it is again unclear if this actually represents adaptive design. Furthermore, since the authors refer to previous studies in which significantly higher doses were tolerated, the methods used appear to have been unnecessary.

## 7. Conclusions

Elements of adaptive design have been in use for decades, but only recently have attempts been made to formally define it. There is still confusion over what constitutes adaptive design, and some less understood designs are controversial. The use of adaptive design in clinical trials is increasingly popular, however, due to the potential for shortened development times and reduced costs. In terms of clinical studies, modifications to a study are generally more acceptable during the exploratory phase than the confirmatory stage. Preclinical studies can be considered an exploratory phase of treatment development and may benefit from adaptive design methods as well. The question is whether or not these benefits are substantial enough. Clinical trials can be expensive due to the high cost of medical care for humans. Therefore, the additional effort and costs involved in implementing adaptive design can be justified by the greater savings associated with increased efficiency. Animal studies, on the other hand, have lower operating costs and more restricted budgets, and it remains to be seen if the gains in efficiency are sufficient to offset the increase in complexity. Increased investment at both the clinical and preclinical level, however, may be justified by the accelerated development of new therapies and improved patient care. For example, stroke is one of the leading causes of death and disability worldwide, and treatment options are currently limited. There is therefore considerable incentive to identify and evaluate new treatments as quickly as possible, regardless of any increased costs in the short term, due to the potential for improved outcome and reduced economic burden in the long term. Perhaps the most important aspect of adaptive design with respect to animal studies is the increased statistical power with small sample sizes, which are common in animal research. Using fewer animals not only reduces costs, but also limits the exposure of animals to harmful procedures, which has ethical benefits.

Other aspects of adaptive design must be taken into account as well, however. The availability of personnel with the appropriate statistical knowledge is an important consideration. The degree of familiarity with adaptive design among reviewers may also affect approval of a study. Some modifications may also be at odds with standard guidelines and even prohibited under current regulations. For example, the Stroke Therapy Academic Industry Roundtable (STAIR) has published guidelines for the conduct of preclinical studies [25]. These guidelines recommend that studies be performed in a blinded fashion. Modifications such as sample size adjustment, however, may require unblinded data, and changes to treatment group assignment may require administrative approval.

To date, adaptive design has been used mainly in clinical trials and received little attention in animal studies. Consideration should be given, however, to evaluating its benefits in a preclinical setting. Formal studies should be conducted to determine the viability of adopting adaptive design, as well as the costs and benefits involved. It may be necessary to establish an infrastructure for education and support on the statistical issues involved. Changes in regulations on animal use may also be required. Although adaptive design has the potential to benefit animal studies, further investigation and discussion is required to determine if it will be ultimately useful.
